# The Effect of Thymus Vulgaris Essential Oil and Chlorhexidine on Candida Albicans Accumulated on Removable Orthodontic Appliance: A Clinical Trial

**DOI:** 10.30476/DENTJODS.2021.89317.1404

**Published:** 2022-06

**Authors:** Navid Naseri, Arefe Kalantari Khandani, Tahereh Baherimoghadam, Azade Kalantari Khandani, Shahram Hamedani, Sadegh Nouripour-Sisakht, Roja Safaeian

**Affiliations:** 1 Dept. of Orthodontic, School of Dentistry, Shiraz Branch, Azad University, Shiraz, Iran; 2 Dentist, School of Dentistry, Shiraz Branch, Azad University, Shiraz, Iran; 3 PhD Student, Dept. of Drug and Food Control, School of Pharmacy, Kerman University of Medical Science, Kerman, Iran; 4 Oral and Dental Disease Research Center, School of Dentistry, Shiraz University of Medical Sciences, Shiraz, Iran; 5 Medicinal Plants Research Center, Yasuj University of Medical Sciences, Yasuj, Iran; 6 Dept. of Natural Resources and Environmental Engineering, School of Agriculture, Shiraz University, Shiraz, Iran

**Keywords:** Orthodontic Appliances, Essential Oil, Candida Albicans, Chlorhexidine

## Abstract

**Statement of the Problem:**

Orthodontic removable appliances can facilitate the accumulation of certain microorganisms and microbial plaque on tooth surfaces and appliance components. Since long-term use of chemical agents is not recommended, an alternative option would be medicinal plants for disinfection of the oral cavity.

**Purpose:**

The aim of this study was to assess the efficacy of *thymus vulgaris* (T. vulgaris) essential oil to decrease the count of *candida albicans* (C. albicans) accumulated on removal orthodontic appliances.

**Materials and Method:**

In this 2-arm parallel controlled clinical trial, forty-four patients whose removable orthodontic appliances were contaminated with C. albicans were randomly divided into two groups by electronic random sampling. In the first group, T. vulgaris essential oil spray and in the second group, 0.2% chlorhexidine (CHX) spray was applied to the appliances. Thereafter, the C. albicans colony count was measured at baseline (T0) and at 2 (T1) and 5 (T2) days after the intervention. Microbial samples were collected by a sterile swab from 3 spots on the internal surface of orthodontic appliances. The Friedman test was used for within-group comparisons and the Mann Whitney test was employed to compare the efficacy of T. vulgaris essential oil with CHX.

**Results:**

The results of this study revealed that continuous use of 2%T. vulgaris essential oil or 0.2% CHX significantly decreased the colony count of C. albicans on removable orthodontic appliances; Howevere, no significant difference was noted in the efficacy of 2% T. vulgaris essential oil and 0.2% CHX in decreasing the C. albicans colony count.

**Conclusion:**

T. vulgaris essential oil and CHX have favorable antimicrobial activity against C. albicans. Moreover, 2% T. vulgaris essential oil can be considered as an alternative to 0.2% CHX to eliminate fungal contamination of orthodontic appliances.

## Introduction

Acrylic removable orthodontic appliances are commonly used for myofunctional therapy or simple tooth movements in children and adolescents during the prepubertal or pubertal stage. Moreover, these appliances are used during the maintenance phase after completion of orthodontic treatment to maintain the results and prevent relapse [ 1 ]. While long-term daily use of removable orthodontic appliances is imperative to achieve ideal treatment results, this would increase the risk of accumulation of microorganisms on the surface of appliances [ 2 ]. 

The constant use of removable orthodontic appliances can facilitate microbial biofilm accumulation on tooth surfaces and other appliance components such as the clasps, springs, and acrylic base [ 3 ]. Microbial biofilm contains different microorganisms that strongly adhere to each other and to the surface; these microorganisms are located in a network of proteins and polysaccharides [ 4 ]. In addition, it is reported that the use of removable orthodontic appliances increases the count of pathogenic microorganisms such as *Streptococcus mutans*, lactobacillus, and C.albicans in unstimulated saliva of patients. Any change in oral microbial flora can increase the risk of caries and periodontal disease [ 5 - 6 ]. 

Evidence shows that use of removable orthodontic appliances can alter the oral microbial flora, This could be related to low salivary flow and accordingly low pH levels, and decreased oral hygiene [ 7 ]. Moreover, the porosities on the internal and external surfaces of the acrylic resin base of such appliances are likely to be covered by pathogenic microorganisms, particularly C. albicans [ 8 ]. The acrylic base of removable orthodontic appliances is made of plastic polymers that contains carbohydrates and possibly can attract several microbial strains such as C. albicans [ 9 ]. 

Chlorhexidine (CHX) is the gold standard for chemical removal of microbial plaque, which is often prescribed as an adjunct to mechanical plaque removal methods. Since long-term use of chemical agents is not recommended, an alternative option would be medicinal plants for disinfection of the oral cavity [ 10 - 11 ]. Several medicinal plants such as thyme, cloves, lavender, and tealeaves have been tested for disinfection of the oral cavity [ 12 , 13 ]. Given that medicinal plants are suitable alternatives to chemical agents, using chemical agents can be limited to acute cases, and herbal products can be recommended for continuous long-term use by orthodontic patients during their treatment course [ 10 ]. 

Thymus (thyme) is an important medicinal herb that belongs to the Lamiaceae family; thymol and carvacrol are the main constituents of T. vulgaris essence, the T. vulgaris essential oil has several pharmaceutical and therapeutic applications [ 14 ]. It also has high chance of acceptance by patients due to its favorable taste and odor, insignificant toxicity and side effects, and low cost [ 15 ]. Evidence shows that Thymus essential oil has suitable antimicrobial and antifungal properties for irrigation of root canals in endodontic treatment *in vitro*, compared with chemical irrigating solutions [ 16 ]. An *in vitro* study verified that T. vulgaris essential oil has favorable antifungal activity in disinfection of removable orthodontic appliances compared to CHX [ 17 ]. The removable orthodontic appliance promotes an increase in salivary Candida carriers particularly albicans species in compare with fixed ones; the removable orthodontic appliance provides more hydrophobic surface to which C. albicans can bind through the hydrophobic effect [ 18 ].

Several *in vitro* studies have shown the effect of different cleaning materalis on removing of C. albicans from acrylic removable dentures [ 17 , 19 - 21 ], but there is no in vivo published study evaluating the procedures used in cleaning removable acrylic orthodontic appliances by comparing the effects of T. vulgaris essential oil with CHX on C. albicans. The aim of this study was to assess the effectiveness of T. vulgaris essential oil with CHX on C. albicans accumulated on removable orthodontic appliances. The null hypothesis stated that the antifungal effectiveness of T. vulgaris essential oil is the same as CHX.

## Materials and Method

### Trial design and any changes after trial commencement

This double-blind, single-center, parallel clinical trial with 1:1 ratio was approved by the Ethics Committee (Reference: IR.IAU.SHIRAZ.REC.1397.004) and registered in the Iranian Registry of Clinical Trials (IRCT20180923041092N1). There were no changes to the trial after its commencement.

### Participants, eligibility criteria and settings

Patients with removable orthodontic appliances with a midline expansion screw whose orthodontic treatment had commenced 6 to 8 months earlier in the Orthodontics Department of School of Dentistry were enrolled in this study. The medical and dental history of patients and their oral health, dental status, type of malocclusion, dental arrangement, and oral hygiene were evaluated before the study. Patients with maxillary transverse deficiency with moderate space deficiency were eligible to enter to this study. The exclusion criteria for patient selection were defined as (1) patients who reported antibiotic intake in the past 3 months, (2) patients who used antimicrobial mouthwashes, (3) patients with very poor oral hygiene (oral hygiene of participants were evaluated by using plaque index and gingival index), (4) those with large diastema or periodontal problem,and (5) patients who suffered from specific nutritional regimens, systemic diseases, syndromic anomalies such as cleft lip or palate and physical or mental disability.

Of 83 candidates for participation in the study, 55 eligible patients were recruited ultimately. The nature and the process of the study were explained to the participants, and then after, written informed consents were obtained from them. 

### Primary sampling to detect patients with C. albicans 

The foremost inclusion criterion for the study was the presence of C. albicans on removable orthodontic appliances of patients. Thus, a primary sampling was performed to ensure the presence/absence of C. albicans on removable orthodontic appliances. For this purpose, a sterile swab dipped in sterile distilled water was used to collect samples from three spots on the internal surface of the removable appliance, which was in contact with the oral mucosa. The three selected regions were at the center of the appliance, close to the gingival margin of teeth #4 and #5, or D and E in the right side, and finally close to the gingival margin of teeth #4 and #5, or D and E in the left side [ 5 ]. The collected sample was streak cultured on a Sabouraud dexterous agar containing chloramphenicol. The plates were coded and sent to a microbiology lab for the microbial culture. Each participant was allocated a code. Of 55 patients, 44 were positive for C. albicans and were enrolled in the study.

In microbiology lab, the collected microbial plates were incubated for 24-48 h. The proliferated colonies of C. albicans were observed on the culture medium after this time period. Lactophenol cotton blue dye, which is a strong dye for diagnostic staining of slides, was used for identification of C. albicans colonies.

### Intervention 

After finding the patients with orthodontic appliances contaminated with C. albicans, patients were randomly divided into two groups based on the application of 2% T. vulgaris essential oil and 0.2% CHX.

In the intervention group, the patients received T. vulgaris essential oil spray. In order to prepare the T. vulgaris essential oil, some research was conducted to find T. vulgaris with higher concentrations of thymol and carvacrol. Thus, T. vulgaris was obtained from Aliabad Tang village in Beyza city of Fars Province, Iran and T. vulgaris essential oil was extracted by distillation. Analysis of the essence was performed by gas chromatography/mass spectrometry (GC-MS; Agilent Technologies-5975C- MS,7890A-GC) to measure the concentrations of thymol and carvacrol. A clinical trial reported the minimum inhibitory concentration (MIC) of T. vulgaris essential oil against C. albicans to be 1.56% (equal to 15.6µL/mL) [ 17 ]. Considering the possibility of procedural errors and to increase the effect size, 2% concentration of T. vulgaris essential oil was formulated in this study; it was collected in 50mL sprays. 

The second group received 0.2% CHX Najo in the form of spray with the same packaging and labeling as those administered for the intervention group. Similarly, subjects were instructed with complete information on how to use the spray and perform daily hygienic measures. The sprays were coded. The patients received comprehensive instructions on how to use the sprays along with their daily oral hygiene. In addition, comprehensive usage instructions were provided on the labels of their sprays. The patients were requested to spray the internal surface of their orthodontic appliance (with15 cm distance) every night after washing their appliance and then rinse orthodontic appliance again after 15 minutes.

### Outcomes (primary and secondary) and any changes after trial commencement 

The second microbial sampling was performed in both groups at baseline prior to the commencement of clinical trial (T0) and at 2 (T1) and 5 days (T2) after using the sprays. A sterile swab was used for sampling from three spots of the appliance as explained earlier for the first sampling. The person in charge of preparing and providing the sprays to the participants and those individuals responsible for culture and diagnosis of the 

slides were blinded to the study. 

### Sample size calculation 

A total of 40 participants were required to achieve 85% power (instituted by G power, version 3.0.1; Franz Faul universitat, Kiel, Germany) and to detect significant differences considering the effect size of 0.47 (*p*< 0.05). In view of the possible dropouts, the sample size was increased to 44.

### Randomization

Before the trial commencement, a researcher who was not involved in the study performed randomization process, allocation concealment, and implementation. Random allocation software program was used for simple electronically-generated randomization. Randomization ensured patients' allocation based on sex and the level of C. albicans colony count data to both groups with 1:1 ratio (Table 1). Double blinding was ensured and only the cooperating chemist was aware of the distribution of disinfectants who confidentially assigned a number for each bottle.

**Table 1:** Classification of colony count data

Classification	Number of colonies
Rare	10 ≥ Number of colonies
Few	10 ≥ Number of colonies >30
Moderate	30 < Number of colonies ≥ 60
Many	60 < Number of colonies ≥ 100
Numerous	Number of colonies > 100

### Statistical analysis 

Data were analyzed using SPSS version 22.0 (IBM, Armonk, NY, USA) using descriptive and inferential statistics. Distribution of data was evaluated using the Kolmogorov-Smirnov test. Since data were not normally distributed, non-parametric tests were applied.

The Friedman test was used to assess the efficacy of T.vulgaris essential oil and CHX within each group. The Mann Whitney test was applied to compare the efficacy of T. vulgaris essential oil and CHX. A value of *p*< 0.05 was considered statistically significant.

## Results

Of 83 patients, 28 patients who did not meet the eligibility criteria were excluded. Of the remaining 55, 44 had removable orthodontic appliances contaminated with C. albicans and were enrolled in the study. 

**Figure 1:**
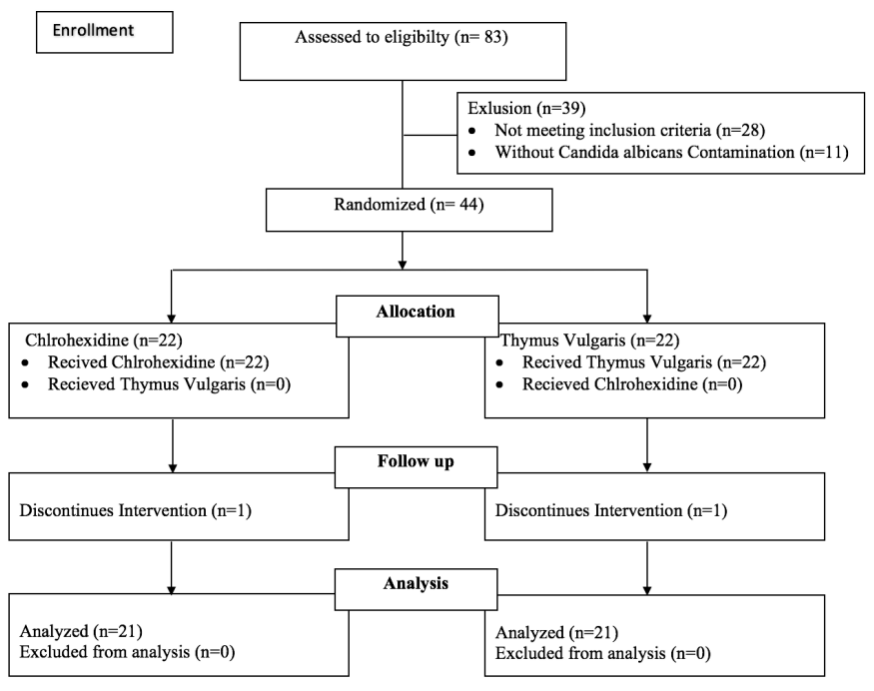
CONSORT flow diagram

Recruitment began in April 2019 and was completed in August 2019. Two participants were later excluded since their orthodontic appliance was broken and they did not cooperate well after the trial commencement, hence, 42 patients completed this trail. The CONSORT diagram is shown in Figure 1. The baseline characteristics for age, sex, and the time point at which patients were recruited were similar in both groups (Table 2).

**Table 2:** Basic characteristics for patients in each groups

	Thymus vulgaris ChlorhexidineThymus vulgaris Chlorhexidine	Thymus vulgaris ChlorhexidineThymus vulgaris Chlorhexidine
Age	10.4±1.9	9.9±1.1
Sex		
Boy	11(52.38%)	13(61.90%)
Girl	10 (47.62%)	8 (38.10%)

### Numbers analyzed for each outcome, estimation and precision, subgroup analysis

For data analysis, the colony count was categorized into five classes of rare, few, moderate, many, and numerous (Table 1). The Friedman test revealed that continuous use of T. vulgaris essential oil or CHX significantly decreased the colony count (*p*< 0.05, Table 3). The Mann Whitney test showed that this reduction was not significantly different between the two groups (Table 3).

**Table 3:** Within-group and between-group comparisons of the mean rank of colony count

	Number	Mean Rank			*p* Value †
		T0	T1	T2	
Thymus Vulgaris	21	2.50	1.87	1.63	0.002**
Chlorhexidine	21	2.60	1.67	1.73	0.001**
*p* Value ‡		0.505	0.809	0.501	

T0: first day of intervention; T1: Two days after intervention; T2: Five days after intervention.

† Mann Whitney test

‡ Friedman test

*: *p*< 0.05; **: *p*< 0.01; ***: *p*< 0.001

**Figure 2:**
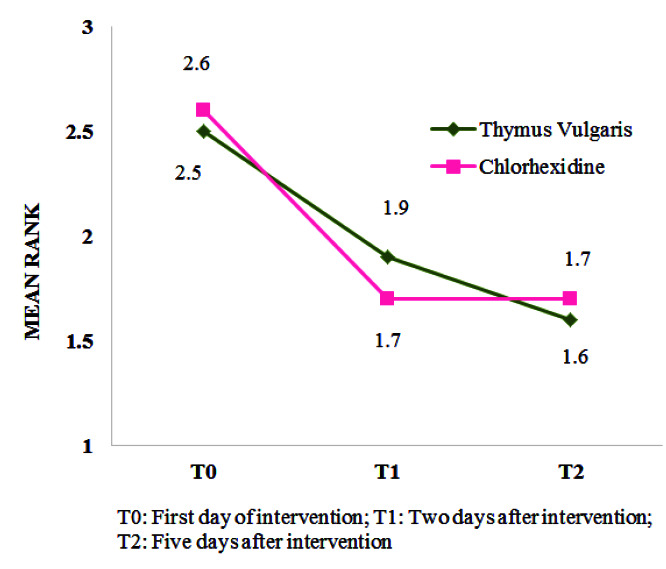
Change in the mean rank of colony count in the two groups

Figure 2 shows the change in the mean rank of colony count (contamination rate) in the two groups. Although the mean difference in the first and third samplings was the same and equal to 0.1, the 2% T. vulgaris essential oil graph was completely descending, while the 0.2% CHX graph reached a plateau from the second phase on. 

**Figure 3:**
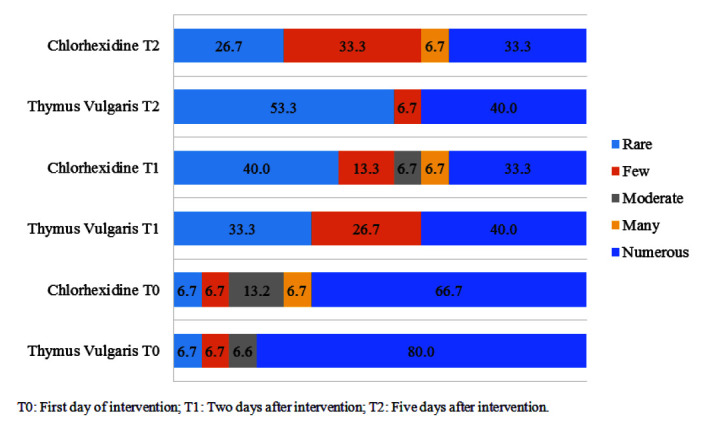
Relative frequency of different levels of *Candida* albicans colony count at different time points

Figure 3  shows the relative frequency distribution of different levels of colony count in the two groups at different time points. The relative frequency of "rare" class was 6.7% in both groups at baseline while the frequency of "rare" class was 53.3% in the 2% T. 

Vulgaris essential oil and 26.7% in the CHX group in the final sampling, which indicated higher efficacy of T. vulgaris essential oil than CHX. The relative frequency of “numerous” class was 80% in T. vulgaris essential oil group at baseline, which decreased to 40% in the final sampling; This rate was 66.7% at baseline in the CHX group, which decreased to 33.3% in the final sampling. In T. vulgaris essential oil group, the remaining percentage mainly belonged to the “few” class in the final sampling. In the CHX group, the relative frequency of “few” and “many” was 40% in the final sampling, with “few” class having a higher frequency. The interpretation of GC-MS results revealed that the essential oil used in this study contained 43.7% thymol. Table 4 lists the 14 main constituents of this essential oil.

**Table 4:** Main constituents of Thymus vulgaris essential oil

Compound	Compound %
Alpha- Phellandrene	1.30
Alpha-Pinene	5.65
Camphene	1.88
Beta-Pinene	0.42
Beta-Myrecene	1.35
Alpha-Terpinene	0.76
Cymene	16.96
Gamma-Terpinene	4.52
Cis-Sabinenehydrate	1.46
Camphor	1.16
Borneol	4.33
Alpha-fenchyl acetate	0.94
Thymol methyl ether	1.77
Thymol	43.7

## Discussion

Orthodontic appliances can impair the balance of micro-organisms in the oral environment and may even lead to disease [ 9 ]. The acrylic base of removable orthodontic appliances is made of polymethyl methacrylate-based auto-polymerizing resin while the wires are made of stainless steel. The pathogenic microorganisms can lodge in resin porosities on the external and internal surfaces of acrylic components; moreover, degradation of acrylic resin can cause bacterial biofilm accumulation [ 22 ].

The mechanical plaque removal methods such as tooth brushing are suitable for elimination of the accumulated biofilm. Nonetheless, mechanical methods alone are not highly effective in many individuals due to poor motivation or hand skills [ 23 ]. Thus, chemical plaque removal agents are always considered along with the mechanical methods. Several chemical and herbal products are used to eliminate or reduce the microorganisms [ 23 ]. CHX is the gold standard for chemical removal of the microbial plaque, nonetheless, CHX is a synthetic chemical agent, which can have many side effects following long-term application [ 11 ] and the long-term use of chemical agents is not recommended [ 10 ]. In the recent years, the demand for alternative plaque removal methods has greatly increased. Herbal products are gaining an increasing popularity due to their organic nature and less side effects [ 14 ]. The results of this clinical trial showed that T. vulgaris essential oil and CHX effectively decreased the C albicans colonies accumulated on orthodontic appliances. 

T. vulgaris is a medicinal plant with antimicrobial properties [ 19 ]. It has favorable taste and odor and low cytotoxicity [ 16 ]. Nonetheless, the in vivo effect of T. vulgaris essential oil on C. albicans isolated from removable orthodontic appliances has not been previously evaluated. According to a study by Kaviani *et al.* [ 17 ] the MIC of T. vulgaris against C. albicans isolated from the surface of acrylic appliances was 1.56% (15.6µL/mL). Thosar *et al.* [ 12 ] reported the MIC of T. vulgaris essential oil to be 1.6% (16µL/mL), with the difference that they used standard-strain C. albicans *in vitro*. Moreover, Fani *et al.* [ 20 ] showed that the MIC of T. vulgaris essential oil was 1.63% (16.3 µL/mL). In this clinical trial, 2% T. vulgaris essential oil was formulated to compensate for the possible procedural errors. 

Although immersion of orthodontic appliances in disinfecting solutions for a certain period of time is the conventional method for disinfection of these appliances at home [ 24 ], immersions can lead to water sorption and subsequent adverse changes in the acrylic resin structure. In this study, the spraying technique was used instead of immersion to prevent possible changes in acrylic resin structure. Also, spraying is cost-effective and easier for patients [ 8 ].

The results of this clinical trial revealed that 2% T. vulgaris essential oil and 0.2% CHX had suitable antifungal effects on orthodontic appliances contaminated with C. albicans. Nonetheless, the reduction in colony count was not significantly different between the two groups. The antimicrobial properties of medicinal plants mainly depend on the presence and concentration of phenolic compounds, saponins, and flavonoids in their structure. These compounds can adversely affect the plasma membrane of microorganisms or inhibit their structural cell membrane enzymes and exert their antimicrobial effects as such [ 25 ].

Thymol and carvacrol are among the main constituents of T. vulgaris essential oil, and the antifungal properties of this plant can be attributed to these ingredients. Carvacrol is an isothymol that increases the activity of ATPase and inhibits the activity of the enzyme responsible for non-specific permeability of the cell membrane of microorganisms such as bacteria, and consequently increases their sensitivity to external factors entering the cell [ 26 ]. Thus, difference in the MIC of T. vulgaris essential oil can be attributed to the variations in the percentage of its constituents.

Assessment of the mean rank-time graph revealed that continuous use of T. vulgaris essential oil during the study period caused a continuous reduction in C. albicans colony count. However, CHX caused a reduction in colony count to some extent and then reached a plateau. In addition, assessment of the relative frequency of C. albicans colonies revealed that T. vulgaris essential oil was more effective than CHX for reduction of the relative frequency of C. albicans colonies. To evaluate the effect of prolonged use of T. vulgaris essential oil on oral mucosa and teeth, enlarging treatment duration is necessary. Further studies on the antimicrobial efficacy of T. vulgaris essential oil against other microorganisms accumulated on orthodontic appliances and dentures are required prior to extensive clinical use of this essential oil as an alternative to CHX. 

## Conclusion

As an alternative to CHX, 2% T. vulgaris essential oil can be suggested to eliminate fungal contamination of orthodontic appliances. In this study we found no significant difference in antifungal efficacy of 2% T. vulgaris essential oil and 0.2% CHX against C. albicans isolated from removable orthodontic appliances.

## Conflict of Interest:

The authors declare that they have no conflict of interest.
